# Clinical implications of the overshoot effect for treatment plan delivery and patient‐specific quality assurance for step‐and‐shoot IMRT

**DOI:** 10.1120/jacmp.v17i4.6129

**Published:** 2016-07-08

**Authors:** John A. Baines, Sylwia J. Zawlodzka, Matthew L. Parfitt, Brigid E. Hickey, Andrew P. Pullar

**Affiliations:** ^1^ Radiation Oncology Mater Centre, Princess Alexandra Hospital Brisbane QLD Australia

**Keywords:** step‐and‐shoot IMRT, overshoot effect, radiotherapy

## Abstract

In this work, overshoot and undershoot effects associated with step‐and‐shoot IMRT (SSIMRT) delivery on a Varian Clinac 21iX are investigated, and their impact on patient‐specific QA point dose measurements and treatment plan delivery are evaluated. Pinnacle^3^ SSIMRT plans consisting of 5, 10, and 15 identical $5\times 5\;{\text{cm}}^{2}$ MLC defined segments and MU/segment values of 5 MU, 10 MU, and 20 MU were utilized and delivered at 600/300 MU/min. Independent of the number of segments the overshoot and undershoot at 600 MU/min were approximately ±10%,±5%, and ±2.5% for 5 MU/segment, 10 MU/segment, and 20 MU/segment, respectively. At 300 MU/min, each of these values is approximately halved. Interfractional variation of these effects (10 fractions), as well as dosimetric variations for intermediate segments, are reduced at the lower dose rate. QA point‐dose measurements for a sample (n=29) of head and neck SSIMRT beams were on average 2.9% (600 MU/min) and 1.7% (300 MU/min) higher than Pinnacle^3^ planned doses. In comparison for prostate beams (n=46), measured point doses were 0.8% (600 MU/min) and 0.4% (300 MU/min) higher. The reduction in planned‐measured point‐dose discrepancies at 300 MU/min can be attributed in part to the inclusion of the first segment (overshoot) in the admixture of segments that deliver measured dose. Pinnacle^3^ plans for 10/9 head and neck/prostate treatments were adjusted by ±0.5 MU to include the effects of overshoot and undershoot at 600 MU/min. Comparing original and adjusted plans for each site indicated that the original plan was preferred in 70% and 89% of head and neck and prostate cases, respectively. The disparity between planned and delivered treatment that this suggests can potentially be mitigated by treating SSIMRT at a dose rate below 600 MU/min.

PACS number(s): 87.55.Qr, 87.56.bd, 87.56.N‐

PACS number(s): 87.55.Qr, 87.56.bd, 87.56.N-

### I. INTRODUCTION

In step-and-shoot IMRT using the 120-leaf MLC system on a Varian Clinac 21iX machine (Varian Medical Systems, Palo Alto, CA) the correspondence of planned and delivered dose/segment is influenced by factors such as leaf positional accuracy, dose rate stability during the "shoot" phase, and beamlet on/off control.[1] As far as the latter is concerned, this is limited by the frequency (~ 13–20 Hz) with which the MLC controller receives information about the delivered MU.[2] This results in a temporal overshoot ~ 50–80 ms[3] and associated overdose for the first segment which is ~ 0.5 MU for 600 MU/min.[4] The linac dose monitoring system

[a] Corresponding author: John A Baines, Radiation Oncology Mater Centre, Princess Alexandra Hospital, 31 Raymond Terrace, South Brisbane 4101, QLD, Australia; phone: (61) 7 3840 3256; fax: (61)7 3840 3294; email: john.baines@health.qld.gov.au tracks the cumulative MU for a given beam and initiates beam termination when the planned MU is delivered.(5) Consequently the overshoot, coupled with less significant MU discrepancies for intermediate segments,(4) causes the last beamlet to be interrupted before the planned MU is delivered, the so-called undershoot effect. The significance of these effects increases with decreasing MU/segment and decreases with decreasing dose rate.(3) For a given dose rate, the magnitude of the overdose and underdose are approximately the same. However, the extent to which they negate in beam integral delivered dose is dependent on the overlap of these segments.

At our institution, Clinac 21iX linacs are used to deliver SSIMRT for head and neck and prostate cancer using 600 MU/min. Treatment plans derived using Pinnacle$^3$ v 9.8 (Philips Medical Systems, Fitchburg, WI) invariably feature multiple ~ 5 MU segments for which overshoot and undershoot effects equate to ~ ± 10% dose delivery discrepancies.(4) For that reason, an investigation of SSIMRT segment delivered dose inaccuracies and their interfractional variation was undertaken. The dose rate dependence of these effects was evaluated using dose rates of 600 MU/min (default value at our facility) and 300 MU/min.

In addition, a detailed investigation of intermediate segment dose variation was performed, as this had not been previously reported.

As is common practice, we compare measured and planned point doses (tolerance ± 3%) for patient specific SSIMRT QA. The impact of segmental dose errors on the differences between them was evaluated at both dose rates as this had not been investigated in previous studies.

Finally, two oncologists reviewed treatment plans for 9 prostate and 10 head and neck treatments, with and without allowance for ± 0.5 MU overshoot and undershoot effect. This extended the work of Kuperman and Lam(4) and demonstrated how Pinnacle$^3$ v 9.8 can be used to clinically evaluate the significance of these effects.

### II. MATERIALS AND METHODS

#### A. Overshoot and undershoot effect

Using Pinnacle$^3$ v 9.8, 6 MV SSIMRT beams consisting of 5, 10, and 15 identical segments were configured with doses of 5 MU, 10 MU, and 20 MU per segment. Each segment consisted of a $5 \times 5$ cm$^2$ MLC defined aperture and a $6 \times 6$ cm$^2$ jaw opening with both centered on the CAX. Intersegment beam hold-off was achieved by moving an out-of-field leaf pair,(4) the duration of this maneuver being sufficient to completely collect charge arising from individual segment exposures. Segmental dosimetric measurements were achieved using a 0.6 cm$^3$ Farmer-type chamber (NE2571) centered on the CAX, 100 SSD, at a depth of 5 cm in plastic water, and a UNIDOS$^{webline}$ electrometer (PTW, Freiburg, Germany) in signal controlled auto-start mode.

Each SSIMRT beam was delivered 10 times to evaluate interfraction dose variations for each segment of a particular beam, and an average chamber reading for each segment was calculated. Control segments identical to a single segment of the SSIMRT beams were used to deliver 5 MU, 10 MU, and 20 MU prior to the relevant step-and-shoot sequence. Average control segment readings for each MU were compared to corresponding segment values and percentage deviations between such readings were used to quantify overshoot and undershoot effects, as well as MU discrepancies for intermediate segments. All measurements were performed at dose rates of 600 MU/min and 300 MU/min.

#### B. Patient-specific QA — point doses

At our institution, point-dose measurements are performed as a part of patient-specific SSIMRT QA.(6) A retrospective review was performed to assess differences between measured and planned point doses for 246 prostate beams and 418 head and neck beams all delivered using 600 MU/min. For a selection of plans, point doses were consecutively measured at both 600 MU/min and 300 MU/min and percentage differences with corresponding planned doses were determined. Measurements were performed in plastic water, depth 5.6 cm, using a PTW

PinPoint chamber (type 31016) and UNIDOS[webline] electrometer according to our patient-specific SSIMRT QA protocol.

### C. Plan comparison

Pinnacle[3] v 9.8 SSIMRT patient treatment plans for 10 head and neck and 9 prostate plans were modified by increasing/decreasing the MU of the first segment/last segment by 0.5 MU whilst keeping the relative weighting of other segments fixed and the overall beam MU unchanged. This MU adjustment corresponds to a representative overdose and underdose of the first and last segments at 600 MU/min, respectively.[(4)] Both adjusted and nonadjusted versions of a given plan were de-identified and evaluated by an oncologist to identify the preferred clinical plan.

## III. RESULTS

### A. Overshoot and undershoot effect

Figure 1 shows the percentage deviation of a given segment average reading from the corresponding control segment (± 1 SD) for a 10 segment SSIMRT beam with a segment dose of 5 MU and dose rates of 600 MU/min and 300 MU/min. Overshoot and undershoot effects are clearly evident and the magnitude of each is approximately ± 10%. Table 1 summarizes the overshoot and undershoot for all combinations of MU and segment number investigated. It is evident that

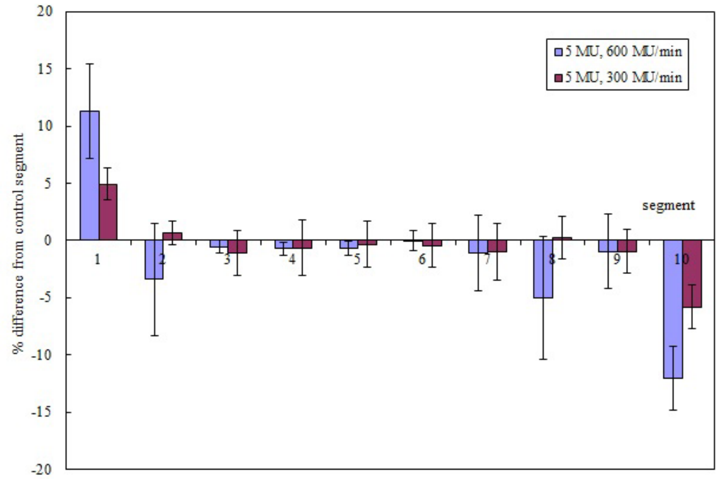

Fig. 1. Average percentage differences from control segment for 5 MU/segment.

Table 1. Average percentage differences (± 1 SD) from control segment for the overshoot and undershoot segments[a].

| | *Segments* | | | | | |
|---|---|---|---|---|---|---|
| | *5* | | *10* | | *20* | |
| *Dose (MU)* | *Overshoot (%)* | *Undershoot (%)* | *Overshoot (%)* | *Undershoot (%)* | *Overshoot (%)* | *Undershoot (%)* |
| 5 | 9.2±2.6 (6.2±1.7) | 13.9±2.1 (6.1±1.6) | 11.3±4.1 (4.9±1.4) | 12.0±2.8 (5.8±1.9) | 11.5±4.0 (5.1±1.2) | 12.6±3.3 (6.1±1.1) |
| 10 | 5.8±1.3 (3.3±0.3) | 7.1±1.2 (2.5±0.7) | 6.5±1.0 (3.5±0.6) | 6.5±1.5 (2.8±0.8) | 5.4±1.1 (3.0±0.6) | 6.1±1.1 (2.6±0.8) |
| 20 | 2.7±0.7 (1.7±0.2) | 3.2±0.7 (1.4±0.3) | 3.2±0.5 (1.7±0.3) | 3.2±0.5 (1.1±0.5) | 2.6±0.7 (1.5±0.4) | 3.1±0.9 (1.2±0.4) |

[a] Values for 300 MU/min are shown in brackets.

the magnitude of these effects is essentially independent of the number of segments (ANOVA $p > 0.05$). Comparison of these effects for 600 MU/min and 300 MU/min indicates that both are reduced by a factor of two at the lower dose rate, which is consistent with previously reported work.[3,4] For a given number of segments and dose rate, the overshoot and undershoot for 5, 10, and 20 MU are approximately in the ratio 1:0.5:0.25 (Table 1). Interfractional variations of overshoot and undershoot at both dose rates are shown in Fig. 2 for a 10-segment 5 MU/segment SSIMRT beam. At the higher dose rate, greater deviations from the mean overshoot and undershoot are observed (Table 1).

Intermediate segments exhibit smaller average percentage differences (Fig. 1) from their respective reference values, as has been reported[4] and do not exhibit the same ~ 2:1 ratio as the overshoot and undershoot effects with decreasing dose rate. For a 10-segment SSIMRT beam, Fig. 3 shows a comparison of the interfraction variations measured for segment 8 at both dose rates. In general, as can be seen from Table 2, both the standard deviation and the percentage difference range are greater at 600 MU/min.

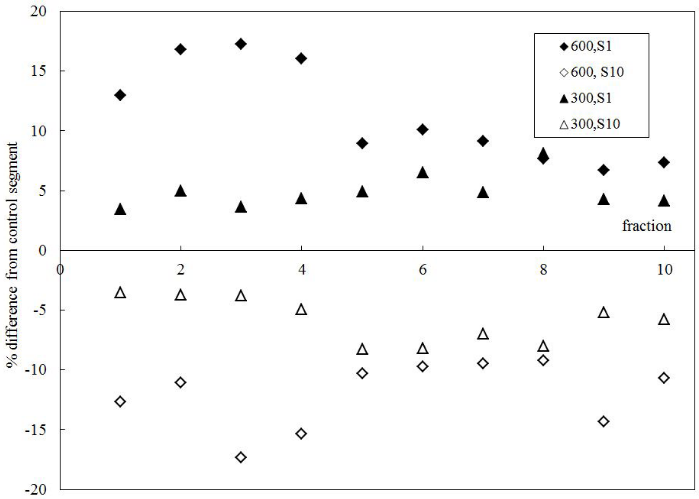

FIG. 2. Interfractional variation of overshoot and undershoot effects for 300 and 600 MU/min for segment 1 (S1) and segment 10 (S10).

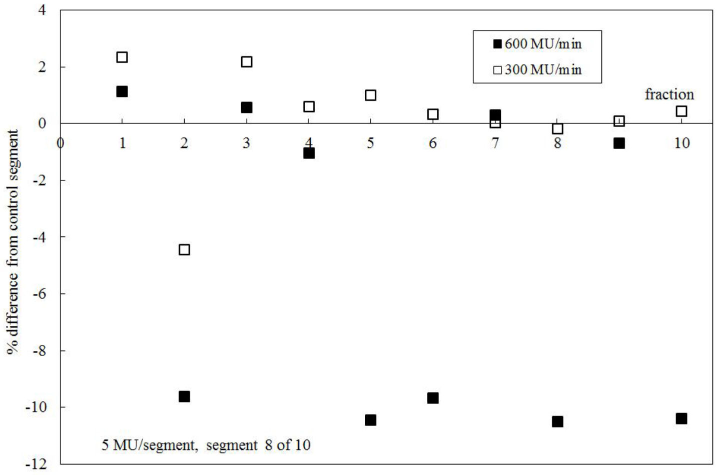

FIG. 3. Example of intermediate segment interfractional variation for 300 and 600 MU/min.

TABLE 2. Standard deviation and range of percentage differences from segment reference values for intermediate segments.

| *Dose rate (MU/min)* | *5 MU/Segment* | *10 MU/Segment* | *20 MU/Segment* |
|---|---|---|---|
| 600 | SD: 3.3%<br>Range: -11.1%–3.1% | SD: 1.5%<br>Range: -5.4%–1.7% | SD: 0.8%<br>Range: -2.6%–0.7% |
| 300 | SD: 1.9%<br>Range: -5.3%–2.4% | SD: 1.1%<br>Range: -2.6%–0.1% | SD: 0.6%<br>Range: -1.1%–0.1% |

### B. Patient-specific QA — point doses

The review of quality assurance measurements, as discussed above, demonstrated that measured dose was systematically larger than planned dose in 76% of head and neck fields and in 71% of prostate fields.

For a sample (n = 29) of the head and neck fields, measured point dose were on average 2.9% and 1.7% higher at 600 MU/min and 300 MU/min, respectively, with a maximum absolute difference between corresponding measured point doses at both dose rates of 4%. In comparison, corresponding differences of 0.8% and 0.4% were observed for prostate fields (n = 46) with a maximum absolute difference of 2.4% between measured point doses at these dose rates.

### C. Plan comparison

Head and neck plans were discriminated on the basis of the conformity index formalism proposed by Paddick[7] and PTV coverage. In 70% of cases the original plan was preferred on the basis of greater conformity index. Figure 4 shows a comparative DVH for one such plan, and it is noted that the PRV SC 5 mm maximum dose for the original/adjusted plan is 44.8 Gy/45.8 Gy. The relevant local dose constraint is 45 Gy and, in as much as the adjusted plan reflects treatment delivery, suggests that this constraint may not be achieved. Figure 5 shows the absolute difference of PTV V95% between adjusted and nonadjusted plans. When the adjusted plan was preferred (plans 2, 4, and 10), it was on the basis of greater PTV coverage, albeit the conformity index was slightly reduced.

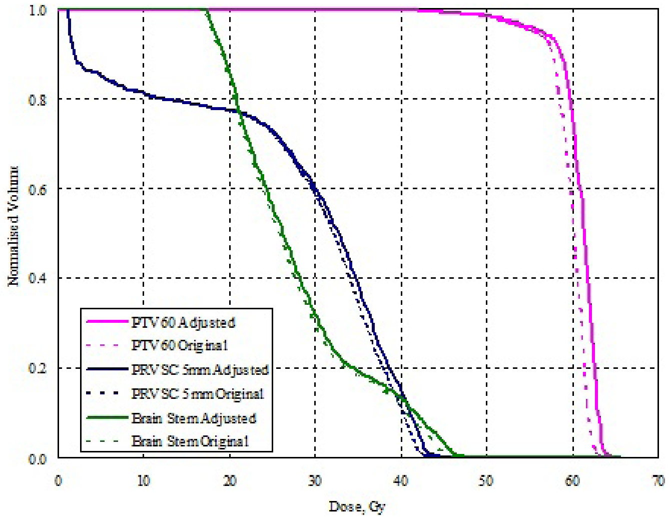

FIG. 4. Example DVH for a head and neck treatment plan with the adjusted plan incorporating an overshoot/undershoot effect.

Prostate plans could not be differentiated using the same definition of conformity index for PTV60/78 and PTV54/66. Instead, preference was determined by dose to the rectum and bladder and, in 89% of cases, the original plan was selected (for one plan the adjusted/nonadjusted plans were equivocal). Rectal dose constraints were V40 < 35% and V65 < 17% for PTV60/78 and V40 < 55% and V65 <35% for PTV54/66. Similarly bladder dose constraints were V40 < 50%, V65 < 25% and maximum dose ≤ 81.9 Gy for PTV60/78 and V40 < 70 %, V65 < 60% and maximum dose ≤ 69.3 Gy for PTV54/66. For all adjusted plans, rectal and bladder doses were higher. Table 3 shows average absolute differences for each dose constraint. Figure 6 shows a representative comparative DVH showing higher dose to the PTV, bladder, and rectum for the adjusted plan.

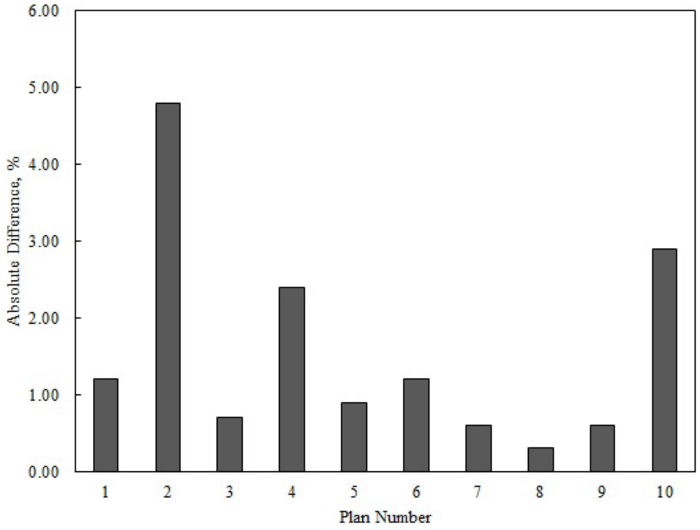

FIG. 5. PTV V95% absolute difference between adjusted and nonadjusted H&N plans.

TABLE 3. Absolute average difference of OAR dose constraints between adjusted and nonadjusted prostate plans.

| *OAR* | *V40 (%)* | *V65 (%)* | *Max Dose (Gy)* |
|---|---|---|---|
| Rectum | 0.6 | 0.9 | - |
| Bladder | 0.3 | 0.7 | 0.4 |

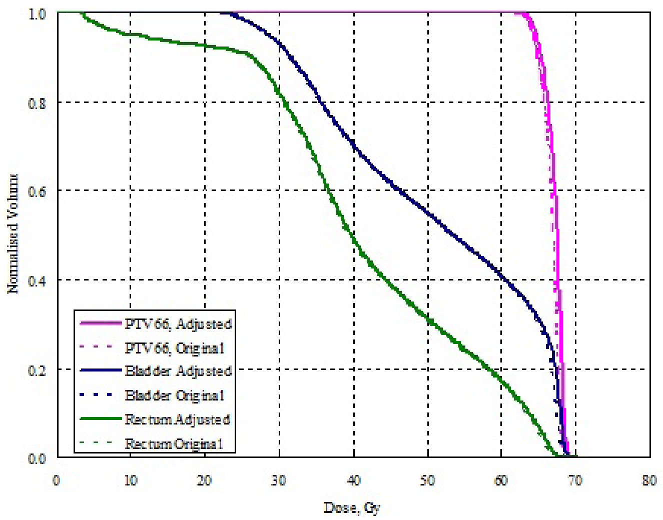

FIG. 6. Example DVH for a prostate treatment plan with the adjusted plan incorporating an overshoot/undershoot effect.

### IV. DISCUSSION

#### A. Overshoot and undershoot effect

With a Clinac 21iX linac there is a temporal mismatch, $\Delta t_i$, between planned and actual beam on time for the ith segment of a step-and-shoot IMRT beam.[3] The average magnitude of this mismatch for the first and last segments (~ 50–80 ms) is significantly greater than that for intermediate segments, giving rise to the so-called overshoot and undershoot effects. In the absence of other factors, the delivered dose discrepancy for the ith segment, $\Delta D_i$ is given by

$$\Delta D_i = \Delta t_i \times R \tag{1}$$

where $R$ is the dose rate.[2] As the dose per segment, $D_i$, is reduced the significance of $\Delta D_i$ increases for a given value of R, as is reported by Kuperman and Lam[4] and observed in this work. From Table 1 it is evident that, independent of the number of segments, the average overshoot effect is approximately 10%, 5%, and 2.5% for MU/segment values 5 MU, 10 MU, and 20 MU, respectively. With the dose rate reduced to 300 MU/min, the overshoot for each MU is halved. For intermediate segments, a delayed beam-on coupled with a delayed beam-off give rise to $\Delta t_i$ values that are generally less than those of the first and last segments.[2] For the last segment, beam delivery is terminated when the linac monitor identifies that the cumulative MU for the SSIMRT beam has been achieved. Dose reduction for the last segment is in large part due to the overshoot of the first segment and is the reason why the overshoot and undershoot effects are of similar magnitude. This is consistent with the observations presented in Table 1 and reported elsewhere.[2] Interfractional variations of the overshoot and undershoot effects are dependent on dose rate (Fig. 2) which is also the case for intermediate segment doses (Fig. 3 and Table 2). Such observations suggest that machine delivery limitations can be mitigated by opting for a reduced dose rate at treatment delivery.

### B. Patient-specific QA — point doses

Figure 7 shows beam's eye view (BEV) examples for the first and last segments of head and neck and prostate SSIMRT fields obtained using the Pinnacle$^3$ v 9.8 IMRT plan optimization algorithm. Clearly the overshoot and undershoot effects of these segments do not negate each other. Further, when selecting suitable points for treatment plan QA, it was found in our review that invariably the admixture of beamlets delivering dose to the PTW chamber included the first but not the last segment. This arises from the open area and weighting attributed to the first segment in the Pinnacle$^3$ optimization of control point sequences for each beam. Given this bias in selection of calculated/measured point doses, the overshoot effect of the first segment will give rise to a dose rate dependent contribution to point dose measurements (Eq. (1)). In addition, the discrepancy between measured and calculated point doses is influenced to some extent by each $\Delta D_i$ in the beamlet admixture contributing to measured dose. However, using dose rates of 600 MU/min and 300 MU/min, it has been reported here that average planned-measured point-dose discrepancies are smaller at the lower rate, albeit that interfraction variations in $\Delta t_i$

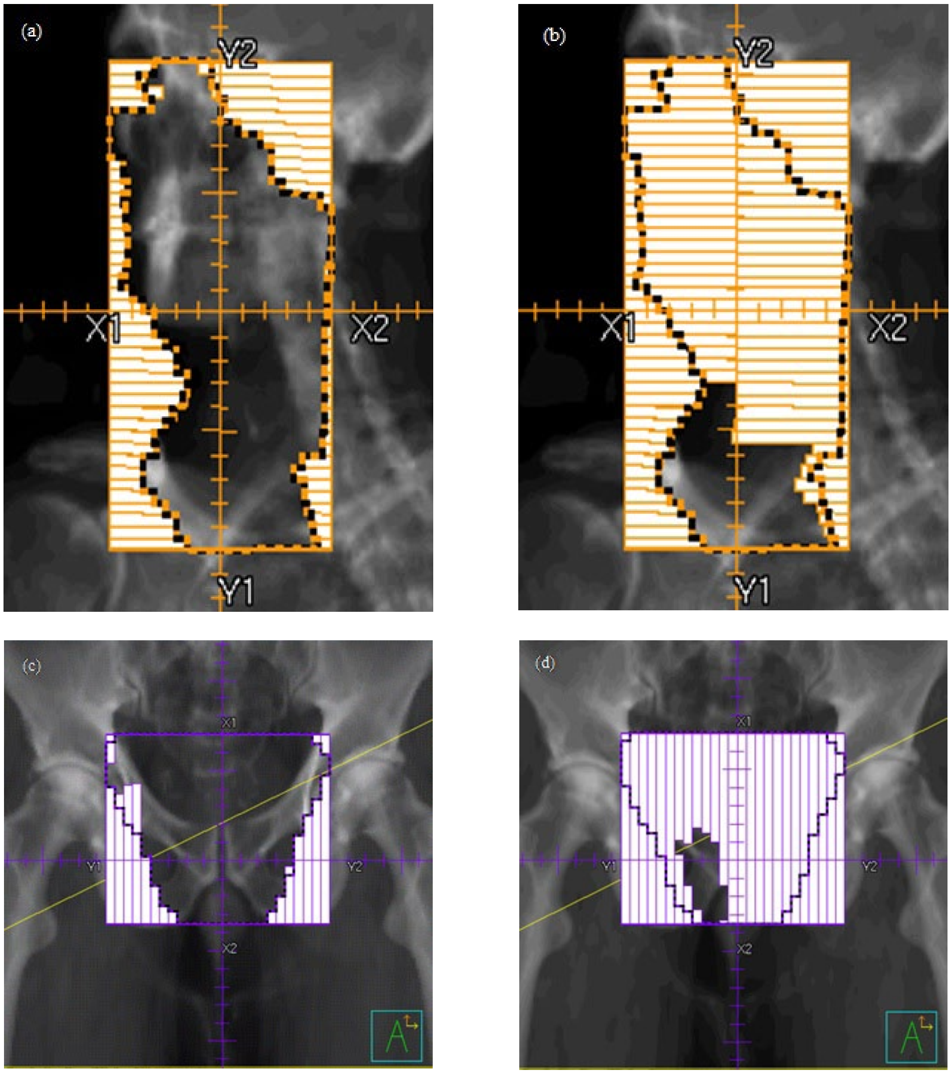

Fig. 7. BEV examples of the first (a) and last segment (b) for a head and neck treatment plan, and the first (c) and last (d) segment for a prostate treatment plan.

impact on measurements at each dose rate for a given point and beam. The average MU for the first segment of the prostate and H&N plans investigated is 19 MU and 8 MU, respectively. Consequently, the percentage overshoot effect is less for prostate plans and thus the dose rate dependence of point-dose discrepancies is less than for H&N plans.

#### C. Plan comparison

In principle, the dosimetric discrepancies associated with $\Delta t_i$ may be reduced by modifying the segment sequences of SSIMRT delivery.[4,5] For example, if the first segment MU is halved and a duplicate segment with the same MU is configured as the last segment of the beam, the overshoot and undershoot effects will negate each other. In practice, such segment manipulation is not a feature of Pinnacle[3] v 9.8. However, it is possible to adjust the MU of the first and last segments whilst constraining the weighting of other segments[5] as is reported in this work. With an increase/decrease of 0.5 MU for the first/last segments, head and neck-adjusted plans, indicative of machine delivery, were inferior to the nonadjusted plans representative of an idealized machine performance. This suggests that the correspondence of planned/delivered treatment is enhanced at a dose rate of 300 MU/min. Reducing the dose rate doubles segment beam on time but, in terms of treatment delivery time that involves intersegment leaf motion as well as gantry rotation, the overall treatment time is not significantly extended at the lower dose rate. For the sample of H&N treatments investigated, the maximum MU/fraction was 755 MU, corresponding to increase in treatment of approximately 80 s.

For the prostate plans investigated, clinical evaluation indicates that nonadjusted plans are preferred on the basis of reduced dose to the bladder and rectum. This further supports the view that, at a reduced dose rate, machine delivery is a better approximation to actual treatment plans.

### V. CONCLUSIONS

When performing patient-specific QA point dose measurements it has been shown that, for the prostate and head and neck plans investigated, greater discrepancies between planned and measured dose occur for the latter. These discrepancies are in part attributed to the inclusion of the first segment in the admixture of segments contributing to measured dose. However, the first SSIMRT segment MU in head and neck plans is significantly less than that for prostate plans. Consequently, the percentage overshoot effect is greater for head and neck plan delivery and greater planned-measured point dose discrepancies are to be expected. Due to the dose rate dependence of the overshoot effect, such discrepancies for head and neck plans decrease more than for prostate plans when the dose rate is decreased.

Interfractional variation of intermediate segment doses is also reduced with decreasing dose rate. For the 10 fractions sampled, at each dose rate it was observed that for varying segments within each fraction the dose discrepancy could intermittently approximate to the overshoot/undershoot for that dose rate. Overall, the temporal errors associated with segment dose delivery in SSIMRT can be mitigated by treatment delivery at the lowest clinically viable dose rate.

Modifications to the MU of the first and last segments of SSIMRT beams is a means to reduce the effects of $\Delta t_i$ and improve the consistency of treatment plans and delivered dose, albeit such an approach ignores the effects of intermediate segment variations $\Delta D_i$. Based on the review of adjusted and nonadjusted plans reported in this work, it is evident that preference clinically was given in large part to the nonadjusted plans. However, the adjusted plans are potentially more indicative of treatment delivery at 600 MU/min. Whilst use of this dose rate may optimize treatment delivery time, operating at a lower dose rate, such as 300 MU/min, improves planned-delivered dose agreement with acceptable increases in treatment time. Further, treatment plans indicating OAR target doses below tolerance levels may in fact be misleading when overshoot effects in treatment delivery at 600 MU/min increase doses above these levels.

### COPYRIGHT

This work is licensed under a Creative Commons Attribution 3.0 Unported License.

## Supporting information

Supplementary Material

Supplementary Material

Supplementary Material

Supplementary Material

Supplementary Material

Supplementary Material

Supplementary Material

Supplementary Material

Supplementary Material

Supplementary Material
