# Supplementary figures and images for: Clinical implications of the overshoot effect for treatment plan delivery and patient‐specific quality assurance for step‐and‐shoot IMRT
Source: J Appl Clin Med Phys. 2016 Jul 8;17(4):114–23. doi: 10.1120/jacmp.v17i4.6129 (PMC5690035; doi:10.1120/jacmp.v17i4.6129)

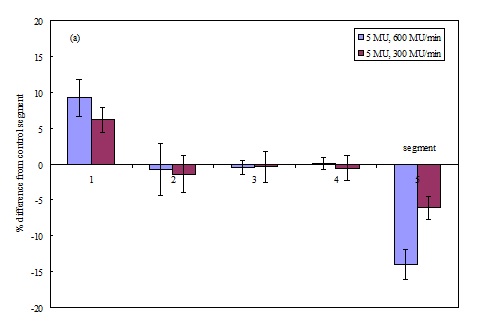

Supplement: Supplementary file 1 — Supplementary Material [file ACM2-17-114-s001.jpg]

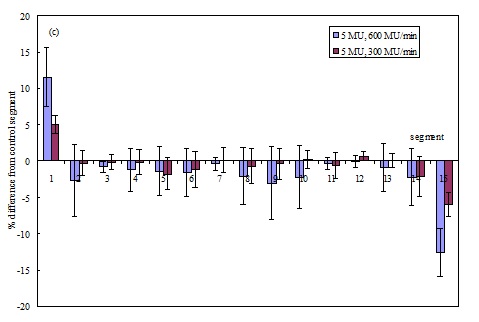

Supplement: Supplementary file 2 — Supplementary Material [file ACM2-17-114-s002.jpg]

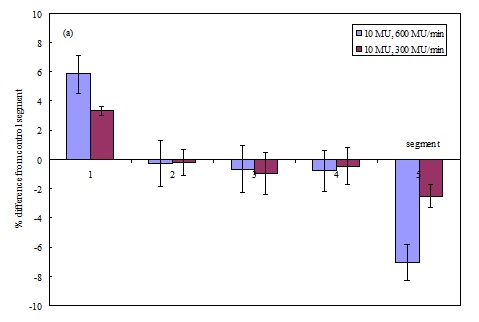

Supplement: Supplementary file 3 — Supplementary Material [file ACM2-17-114-s003.jpg]

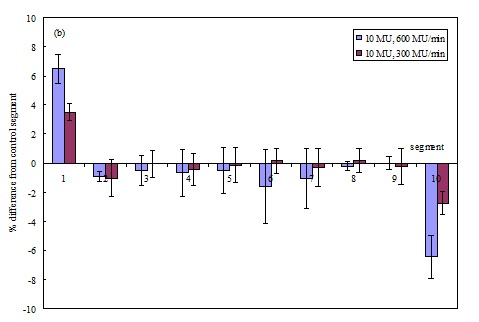

Supplement: Supplementary file 4 — Supplementary Material [file ACM2-17-114-s004.jpg]

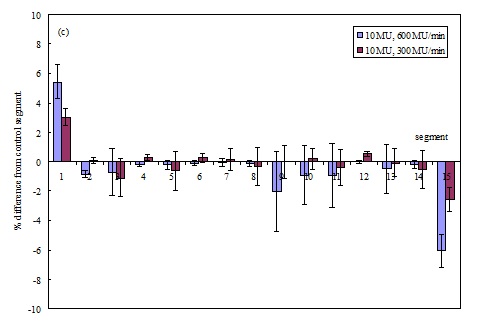

Supplement: Supplementary file 5 — Supplementary Material [file ACM2-17-114-s005.jpg]

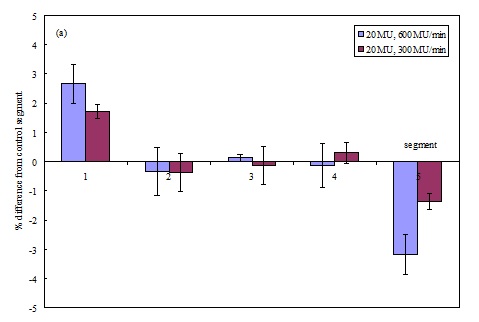

Supplement: Supplementary file 6 — Supplementary Material [file ACM2-17-114-s006.jpg]

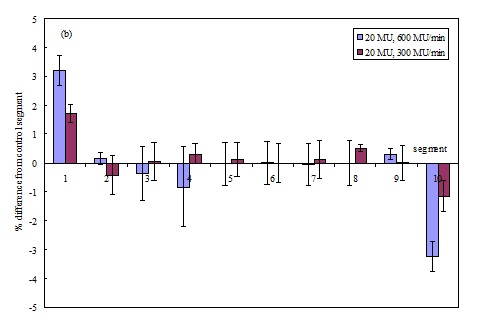

Supplement: Supplementary file 7 — Supplementary Material [file ACM2-17-114-s007.jpg]

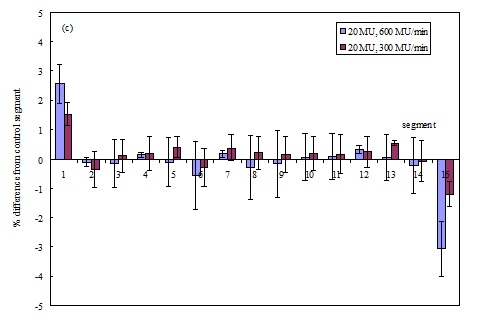

Supplement: Supplementary file 8 — Supplementary Material [file ACM2-17-114-s008.jpg]

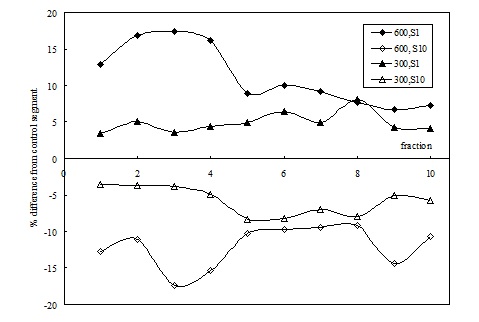

Supplement: Supplementary file 9 — Supplementary Material [file ACM2-17-114-s009.jpg]

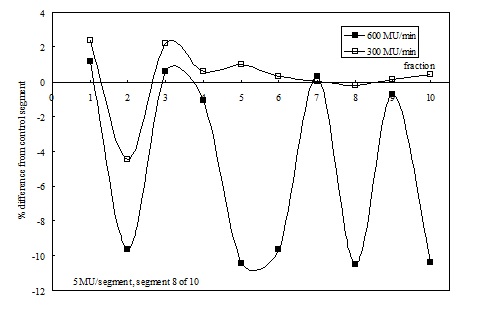

Supplement: Supplementary file 10 — Supplementary Material [file ACM2-17-114-s010.jpg]
